# Continuum of maternity care among rural women in Ethiopia: does place and frequency of antenatal care visit matter?

**DOI:** 10.1186/s12978-021-01265-x

**Published:** 2021-11-06

**Authors:** Tegene Legese Dadi, Girmay Medhin, Habtamu Kebebe Kasaye, Getnet Mitike Kassie, Mulusew Gerbaba Jebena, Wasihun Adualem Gobezie, Yibeltal Kiflie Alemayehu, Alula Meresa Teklu

**Affiliations:** 1 College of Medicine & Health Science, School of Public Health, Hawassa University, Hawassa, Ethiopia; 2 MERQ Consultancy PLC, Addis Ababa, Ethiopia; 3 Aklilu Lemma Institute of Pathobiology, Addis Ababa University, Addis Ababa, Ethiopia; 4 College of Medicine & Health Science, Department of Midwifery, Wollega University, Nekemte, Ethiopia; 5 International Institute for Primary Health Care – Ethiopia, Addis Ababa, Ethiopia; 6 Institute of Health Science, Jimma University, Jimma, Ethiopia; 7 Averting Maternal Death and Disability (AMDD), Columbia University, New York, NY USA

**Keywords:** Maternal health services, Intensity of continuum of care, Health Extension Program, Place of first ANC visit, ANC visit as per MOH recommendation, Health post

## Abstract

**Introduction:**

The ministry of health (MOH) of Ethiopia recommends 4 or more focused antenatal care (ANC) visits at health centre (HC) or at a higher level of health facility (HF). In Ethiopia, few studies investigated time dimension of maternal health continuum of care but lack data regarding place dimension and its effect on continuum of care. The aim of this study is to estimate effect of place of ANC-1 visit and adherence to MOH’s recommendations of MOH for ANC visits on continuum of care rural in Ethiopia.

**Methods:**

We used data collected from 1431 eligible women included in the National Health Extension Program (HEP) assessment survey that covered 6324 households from 62 woredas in nine regions. The main outcome variable is continuum of care (CoC), which is the uptake of all recommended ANC visits, institutional delivery and postnatal care services. Following descriptive analysis, Propensity Score Matching was used to estimate the effect of place of ANC-1 visit on completion of CoC. Zero inflated Poisson regression was used to model the effect of adherence to MOH recommendation of ANC visits on intensity of maternal health continuum of care.

**Result:**

Only 13.9% of eligible women completed the continuum of care, and place of first antenatal care (ANC) visit was not significantly associated with the completion of continuum of care (β = 0.04, 95% CI = -0.02, 0.09). Adherence of ANC visit to the MOH recommendation (at least 4 ANC visits at higher HFs than health posts (HPs)) increased the likelihood of higher intensity of continuum of care (aIRR = 1.29, 95% CI: 1.26, 1.33). Moreover, the intensity of continuum of care was positively associated with being in agrarian areas (aIRR = 1.17, 95% CI: 1.06, 1.29), exposed to HEP (IRR = 1.22, 95% CI: 1.16, 1.28), being informed about danger signs (aIRR = 1.14, 95% CI: 1.11, 1.18) and delivery of second youngest child at HF (IRR = 1.16, 95% CI: 1.13, 1.20). Increasing age of women was negatively associated with use of services (IRR = 0.90, 95% CI: 0.87, 0.94).

**Conclusion:**

Completion of maternal health continuum of care is very low in Ethiopia, however most of the women use at least one of the services. Completion of continuum of care was not affected by place of first ANC visit. Adherence to MOH recommendation of ANC visit increased the intensity of continuum of care. Intensity of continuum of care was positively associated with residing in agrarian areas, HEP exposure, danger sign told, delivery of second youngest child at health facility. To boost the uptake of all maternal health services, it is crucial to work on quality of health facilities, upgrading the infrastructures of HPs and promoting adherence to MOH recommendations of ANC visit.

## Plain language summary

Maternal health continuum of care is an integrated service delivery of antenatal care, facility delivery and postnatal care on appropriate time and place. Continuum of care averts more maternal mortality than individual service provision. In Ethiopia a small percent of women complete continuum of care. Previous studies in Ethiopia explored the effect of time on CoC, however the effect of place of service delivery on subsequent continuum of care were not addressed. This study, therefore, assessed the effect of place of service delivery on completion of subsequent continuum of care using data from 1431 fixed cohort of women during the National HEP assessment survey.

The proportion of women who took all essential maternal health services was very low. Whether first antenatal care is at health post or at health centre did not have a significant effect on the completion of maternal health continuum of care. Adherence to the existing recommendations of Ministry of Health for antenatal care visit increases the uptake of maternal health continuum of care. Better completion of maternal health services was observed in agrarian than pastoralist areas, among those who have exposure to health extension program, who have information about danger signs, and who deliver their previous child at health facility. We can conclude that good implementation of the health extension program, and improving service quality at health facilities increase the uptakes of maternal health service. Moreover, promoting adherence of women to the ministry of health ANC recommendations increase uptakes.

## Introduction

Globally, improving the lives of mothers has been an important public health and development agenda [1] mainly because of three reasons: firstly, it is a means to minimise the disparity of maternal mortality between developed and developing countries by improving the existing situation in developing countries [2]; secondly, it enables women to lead fulfilling and productive lives by providing support during the pregnancy and postpartum periods [3]; thirdly, improved uptake of maternal health services help to reduce maternal and child morbidity and mortality. Maternal death is associated with increased risk of child death, and negatively impacted economic development [3–5]. World Health Organization (WHO) put a global target to reduce Maternal Mortality Ratio (MMR) to less than 70/100,000 live births (LBs) by 2030 [6]. Accordingly, the Ethiopian government has set a target to reduce it to 199/100,000 LBs by the end of 2020 and 42/100,000 LBs by the end of 2035 through provision of improved maternal health services [7, 8].

Antenatal care (ANC) service during pregnancy, skilled birth attendant (SBA) during delivery, and postnatal care (PNC) service during postpartum period are the major components of maternal health services [9]. The provision of those services as a continuum has been promoted at a global level to improve maternal and child health which in turn reduces morbidities and the death toll occurring every year [9, 10]. Continuum of care (CoC) is defined as integrated service delivery of the above-mentioned services for mothers and children on appropriate time and place [11, 12].

CoC has two important dimensions: the time of care delivery (i.e. from pregnancy to postpartum period), and place of service delivery or level of care delivery [12, 13]. Most of maternal and neonatal deaths occur during the time of delivery and immediate postpartum, when the services are not given in the appropriate health facilities or if it is provided in a poorly equipped health facilities. Most maternal death in Ethiopia occurs at home during postpartum period, which is shoved up by high home delivery [14, 15]. CoC can avert 71% of MMR but reduced to 37% if one of the services is missing from the continuum [16, 17].

Even though CoC is a core principle and framework to save the lives of mothers and babies, there is high drop out of mothers in the continuum as we go from ANC to PNC in Ethiopia [18, 19]. Despite the high proportion (74.0%) of ANC-I attendance in Ethiopia, only 43.0% of women received ANC-4 + , 48% of women delivered at health facility and only 34% of women received a PNC check-up [19]. In other studies in Ethiopia only 9–12% of women completed continuum of care [20, 21]. Even though health centers, health posts and hospitals have different standards in terms of infrastructure and equipment, Health centers and health posts have low maternal health service readiness compared to hospitals due to lack of competent professionals, lack of essential equipment, supplies, and infrastructure [22–24]. This compromised quality of facilities is among the factors that deter women from completion of CoC [25]. The Ethiopian ministry of health (MOH) recommends to have 4 or more ANC visits at the health centre or at a higher level health facility. Although the MOH recommends health posts not to provide focused ANC [26], almost one third of women attend their ANC at the health post [27].

Some of Ethiopian studies investigated time dimension of maternal health continuum of care but lack data regarding place dimension of the CoC [20, 28]. There is paucity of evidence that shows effect of place and frequency of ANC visits as per the recommendations of MOH on subsequent continuum of care. The findings from this study will have significant policy implications to improve maternal health service utilization in Ethiopia and similar settings. The aim of this study is to estimate effect of place of ANC-1 visit and adherence to the recommendations of MOH for ANC visit on CoC using data collected from a fixed cohort of women in Ethiopia using data from the National Health Extension Program assessment [29].

## Methods

### Study setting and context

Ethiopia is located in the Eastern part of Africa, which is administratively divided into five agrarian (agriculture as the main way of living) regions, two pastoralist (livestock raising as the main way of living), two regions with both agrarian and pastoralist areas, and two City administration at time of data collection. Each region was further administratively divided into zones, then into woredas, and finally into kebeles. Kebeles are the lowest government administrative unit and it has an average household size of 500–1000 and a population of 2500–5000. The country has an estimated 100.8 million population of which four-fifth of the population resides in rural settings, with a 4.7 average family size, and a 2.6% average annual population growth rate. Females constitute around 49.8% of the national population and half of these females are within the reproductive age [30].

Maternal health services are delivered in a three-tier health system that includes primary, secondary, and tertiary levels. At the primary level of health service delivery there are health posts staffed with Health Extension Workers (HEWs), health centers staffed with nurses and health officers, and primary hospitals. The health extension program encompasses health posts (HPs) and HEWs, and it is the main service delivery modality at the primary level for the community [31].

#### Data source and its descriptions

We used data from the National HEP assessment survey which field data collection was conducted from March to May 2019. The rural component of the survey covered 62 woreda distributed across all the 9 agrarian and pastoralist regions using multistage sampling design. Three kebeles per woreda, and 34 households (HHs) per kebele were randomly selected. A total of 6324 HHs were recruited from 185 kebeles for the survey. Thus the data is collected from health posts and HHs from the selected kebeles. The respondents for the HH survey were women and their husbands, and HEWs for health post survey [29].

#### Sample size

The study population includes women of reproductive age (15–49 years) who delivered a child in the last two years. The study included 1431 women from 6324 HHs who were part of a fixed cohort from antenatal to postnatal care and the catchment health posts in the selected kebeles. The selected women were asked about their use of maternal health services for their last delivery in the last two years. The health post assessment includes service availability, equipment and human resource characteristics.

### Measurements

#### Outcome of interest

Two outcome of interest were analysed in this paper:

1. **Completion of Continuum of care (CoC):** It is defined by the completion of all recommended ANC visits (at least 4 ANC), institutional delivery (ID) and PNC services. A woman is said to have completed CoC, coded as “1”, if she received all mentioned services, and incomplete CoC, coded as “0”, if she missed at least one of the recommended services. This outcome was used to see effect of place of first ANC visit on continuum of care
2. **Intensity of Continuum of care**: A woman may have a score of 0–6 based on service uptake of ANC-I to ANC-IV, ID and PNC. If a woman utilized all of the services the score is 6, if she did not get any service she gets the score of 0. This outcome was used to investigate effect of ministry of health (MOH) recommendations of ANC visit on continuum of care.

#### Exposure variables

There are two main exposure variables.

1. Place of ANC-1 visit: if the ANC-1 visit was at health post (HP) it is coded as “0” and if it was at health centre (HC) or other higher health facilities (HFs) it was coded as “1”. It will be used to explain completion of continuum of care.
2. ANC visit as per the recommendation of MOH: defined based on 4 or more ANC visits at HC or other higher HFs other than HPs. If a woman has 4 or more ANC visits at HC or other higher HFs, it is coded as “Recommended place and frequency,” and if one of the criteria is not fulfilled, it is marked as “Not recommended place and frequency.” [26]. It will be used to explain intensity of continuum of care.

#### Other covariates

These variables include individual, household, and kebele level factors. Individual level factors include: age, marital status, whether she is a household head or not, family size, maternal and paternal education (whether the mother or husband attended grade one or more education coded as formal education), if she has exposure to HEP (Yes response if a woman took any services at a HP or if she is visited by a HEWs at her home, and No response if she is not visited anywhere), having access to media, awareness of husband and wife about MHS (ANC, ID and PNC) availability, if she was told about danger sign, and place of delivery for the previous child. Household level factors included: wealth index which is categorized into three groups (high, medium and low). The kebele level variables are the number of medical equipment at HP (a continues variable ranging from 0 to 11 which measures the availability of 11 essential medical equipment at the HP like different guidelines, statoscope, BP apparatus …), and human resource at HP which is categorized as “Presence of at most level-3 HEWs” if HPs have level 3 or below HEWs and “Presence of at least level-4 HEWs” if the HPs have at least level 4 HEWs. Access to health facility it is categorized as “Accessed HP/HEW” if the women have a nearby HP or HEW for use and “Accessed other HF” if the women have a nearby health centre or other higher level health facility.

### Conceptual framework

**Fig. 1:**
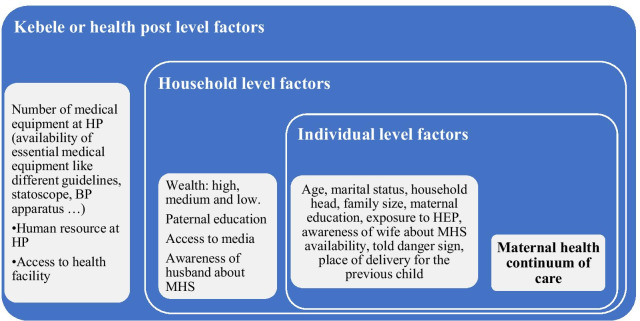
The conceptual framework showing the multilevel factors affecting maternal health continuum of care

We have adapted the social ecological model since the model considers the complex interplay of multiple levels factors and interactions between individuals, household and kebele or health post level factors [32], which will affect the utilization of maternal health service in the continuum (Fig. 1).

### Data management and analysis

We cleaned and analysed the data using Stata version 16.1. We did weighed analysis to account for disproportionate stratification of number of different regions, use of multistage sampling to recruit study participants and to be able to generalize the finding to the national reference population. Wealth quintiles were used as a proxy measure of socio-economic status. Descriptive analysis including frequencies, crosstabulations, and graphical presentations were used to summarize characteristics of study participants across different characteristics. We used Propensity Score Matching (PSM) analysis, which is one of the treatment effect model in stata, to estimate the effect of place of ANC-1 visit on the completion of CoC which is the binary response. We used Zero inflated Poisson (ZIP) regression to model the effect of adherence to MOH recommendation of ANC visits on intensity of maternal health continuum of care that has values ranging from 0 to 6. The model is selected after checking the validity of required assumptions [33]. The exposure variable (adherence to MOH recommendation) has some degree of overlap with the outcome variable, intensity of CoC. We have conducted re-analysis of the model after removal of the overlap. We reported the findings as statistical significant whenever p-value was less than 5%.

## Result

### Characteristics of study participants

This finding is based on data collected from a total of 1431 fixed cohort of women. Their mean age was 28.6 years (SD = 6.39, minimum age = 16 years, and maximum age = 48 years), 4% of the participants were from pastoralist areas, majority do not have formal education and 5.8% of the HHs were female headed (Table 1).

**Table 1:** Socio-economic characteristics of study participants

Characteristics of study participants	Unweighted number (n = 1431)	Weighted %
Women education		
No formal education	947	58.96
Formal education	484	41.04
Husband education (n = 1184)		
No formal education	561	38.67
Formal education	623	61.33
Marital status		
Currently married	1342	96.36
Others	89	3.64
Livelihood		
Women in Pastoralist	422	3.98
Women in Agrarian	1009	96.02
Family size		
Up to 4 peoples	437	33.31
5–8 peoples	854	55.2
9 or more peoples	140	11.49
Wealth index		
Lower quintile	407	25.51
Middle quintile	478	32.68
Higher quintile	546	41.81
Head of HH		
Male headed HH	1310	94.24
Female headed HH	121	5.76
Media		
Have no TV or Radio	938	57.08
Have TV or Radio	493	42.92
Accessed health facility in the last one year		
Accessed other HF	152	7.42
Accessed HP/HEW	1279	92.58

*HHs* Households, *TV* television, *HF* health facility, *HP* health post, *HEW* health extension worker

### Exposure to HEP and awareness about service availability at HP

The mean availability score of medical equipment was 7.98 (SD = 2.98, minimum number = 0, and maximum = 14) and 1.44% of the HPs do not have any medical equipment. More than 80% of the women had exposure to HEP, availability of delivery service at the HP was least known by the women and their husbands, and the commonest known service at HP was ANC. More than 73% of HPs have at least one level IV HEWs (Table 2).

**Table 2:** Exposure to HEP and service awareness about service availability at HP

Characteristics of study participants	Unweighted number (n = 1431)	Weighted %
HEP exposure		
No	332	18.97
Yes	1099	81.03
ANC service awareness at HP		
Both (husband & wife) are not aware	279	10.98
At least one of them aware	414	24.66
Both (Husband & wife) are aware	738	64.35
Delivery service awareness at HP		
Both (Husband & wife) are not aware	747	44.41
At least one of them aware	331	21.73
Both (Husband & wife) are aware	353	33.87
PNC service awareness at HP		
Both (Husband & wife) are not aware	546	33.27
At least one of them aware	405	25.8
Both (Husband & wife) are aware	480	40.92
MHS (ANC, Delivery & PNC) awareness by husband and wife at HP		
Both are not aware for all MHS	242	8.88
At least one of them aware	888	62.21
Both are aware for all MHS	301	28.92
Human resource at the HP		
Have level 3 or below HEWs	526	26.88
Have at least one level 4 HEWs	727	73.12

*HEP* health extension program, *HP* health post, *ANC* antenatal care, *PNC* postnatal care, *MHS* maternal health services, *HEW* health extension worker, *HEWs* health extension worker

More than half of (55.5%) the women were not told at least one danger sign on their previous pregnancy and two third (64%) of women delivered their second youngest child at home (data not shown).

### Maternal health services uptake

**Fig. 2:**
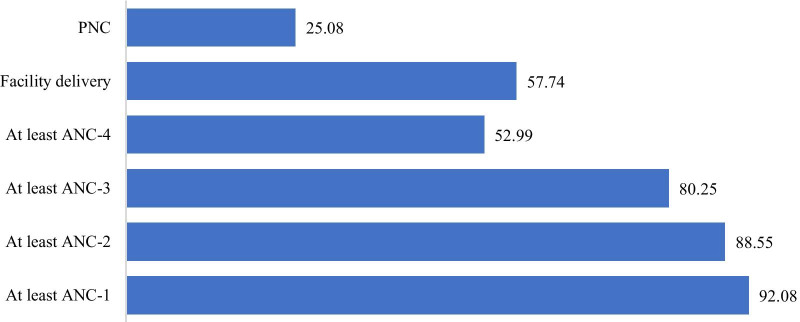
Maternal health service uptake among fixed cohort of women in Ethiopia

Maternal health service uptake decreases as they progress from ANC-I to PNC. For example most of the women (92%) took at least one ANC visit, but only 25% took PNC (Fig. 2).

**Fig. 3:**
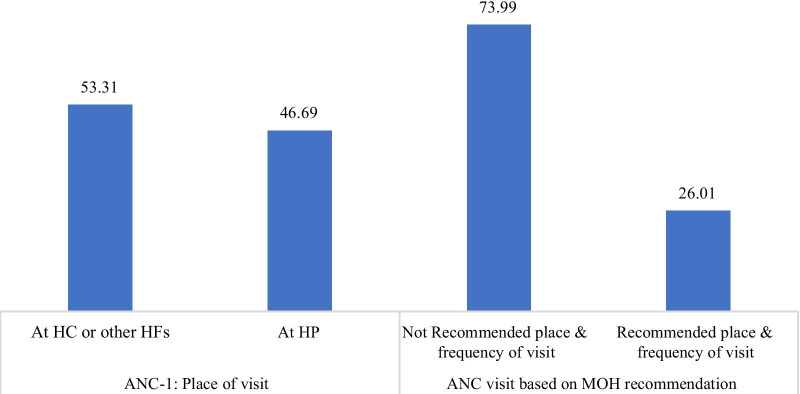
Place of ANC-1 and ANC visits based on MOH recommendation women

Among the study participant women, 47% took their ANC-I at HP and the rest took the services at HC or other higher level HFs. Around three fourth (73.90%) of the women didn’t take all ANC visits based on the recommendation of MOH and only 26.10% have recommended number and place of ANC visits (Fig. 3).

### Place and adherence to MOH recommendation of ANC visits, and maternal health CoC

**Fig. 4:**
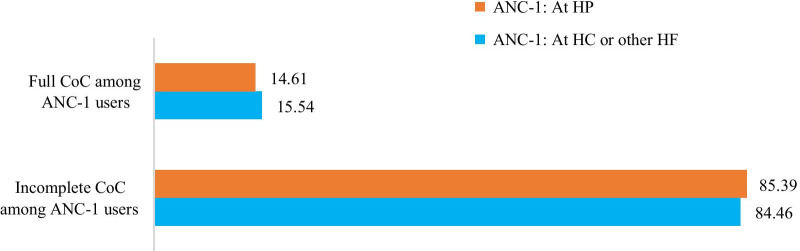
Completion of CoC among women who took their ANC-1 at HP and HC or other higher HFs

Among women who have at least ANC-1, 14.8% completed CoC with no significant difference among women who took their ANC-1 at HP and those who took at HC or other HFs (Fig. 4).

**Fig. 5:**
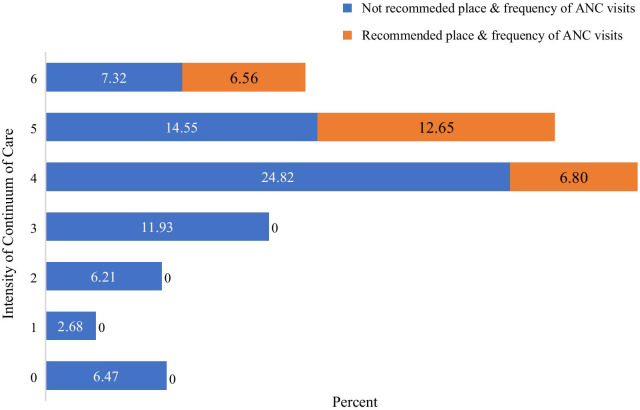
Intensity of maternal health CoC by MOH recommendation of ANC visits among women

There is high drop out of women receiving maternal health services as we go along in the continuum of care. Only 13.88% of the cohort completed the continuum of care, 6.6% of them received MOH recommended ANC visits, 6.5% of women didn’t take any one of the services (Fig. 5).

### Effect of place of ANC-1 visit on continuum of care

The result of PSM showed that place of ANC-1 visit does not have a significant effect on the completion of continuum of care (β = 0.04, 95% CI = − 0.02, 0.09) after adjusting for covariates. The model was adjusted for age, women education, livelihood, family size, wealth index, gender of HH head, exposure to HEP, MHS service awareness by wife and husband, whether she was informed or not about danger sign during ANC visit, status of facility delivery of second youngest child, presence of medical equipment in the HP, status of access to HP, human resource of HP, paternal formal education and media (table not shown).

### The effect of adherence to MOH recommendation of ANC visit on intensity of CoC

Adherence to MOH recommendation of ANC visit was consistently associated with increased intensity of CoC or increased uptake of CoC across all hypothesis testing models. In the fully adjusted model it increases the incidence of uptake of continuum of care by 1.28 times (aIRR = 1.28, 95% CI: 1.24, 1.32) (Table 3).

**Table 3:** A hypothesis testing that shows effect of MOH Recommended ANC visits on CoC among women

Effect of ANC visit based on MOH recommendation	IRR (95% CI)
Crude effect of ANC visit based on MOH recommendation	1.29 (1.26, 1.33)
*Adjusted effect of ANC visit based on MOH recommendation	1.28 (1.24, 1.32)

*Adjusted for: exposure status to HEP, maternal age, women formal education, MHS service awareness by wife and husband, danger sign told, facility delivery of second youngest child, wealth index, family size, wife headed HH, livelihood, number of medical equipment, accessed HP, human resource of HP, and media

### Factors affecting intensity of continuum of care

The intensity of continuum of care increases with exposure to HEP (aIRR = 1.22, 95% CI: 1.16, 1.28), residing in agrarian areas (aIRR = 1.17, 95% CI: 1.06, 1.29), who heard about danger signs (aIRR = 1.14, 95% CI: 1.11, 1.18) and who deliver their second youngest child at HF (aIRR = 1.16, 95% CI: 1.13, 1.20) (Table 4).

**Table 4:** Factors associated with intensity of continuum of care among fixed cohort of women in Ethiopia

Background characteristics		Crude IRR (95% CI)	Adjusted IRR (95% CI)
Adherence to recommendation of MOH to ANC	No	Ref	Ref
	Yes	1.29 (1.26, 1.33)	1.28 (1.24, 1.32)
Exposure to HEP	No	Ref	Ref
	Yes	1.11 (1.07, 1.15)	1.22 (1.16, 1.28)
Age		1.00 (0.99, 1.00)	1.01 (1.00, 1.01)
Women education	No formal education	Ref	Ref
	Have formal education	1.09 (1.07, 1.12)	1.05 (1.02, 1.08)
MHS awareness by husband and wife at HP	Both are not aware for all MHS	Ref	Ref
	At least one of them aware	1.01 (0.96, 1.05)	1.06 (1.00, 1.12)
	Both are aware for all MHS	0.94 (0.89, 0.98)	0.96 (0.96, 1.08)
Told at least one danger sign	No	Ref	Ref
	Yes	1.17 (1.14, 1.19)	1.14 (1.11, 1.18)
The second youngest child place of delivery	Home or other place	Ref	Ref
	Health institution	1.18 (1.15, 1.22)	1.16 (1.13, 1.20)
Wealth index	Lower quintile	Ref	Ref
	Middle quintile	0.94 (0.91, 0.97)	0.91 (0.88, 0.95)
	Higher quintile	1.03 (1.00, 1.06)	0.96 (0.92, 1.01)
Family size	≤4 peoples	Ref	Ref
	5–8 peoples	0.97 (0.95, 0.99)	1.01 (0.97, 1.05)
	≥ 9 peoples	0.88 (0.84, 0.92)	1.02 (0.96, 1.08)
Head of HH	Husband headed HH	Ref	Ref
	Wife headed HH	1.04 (0.98, 1.09)	0.97 (0.91, 1.03)
Number of medical equipment		1.01 (1.00, 1.01)	1.01 (1.01, 1.02)
Media	Have no TV or Radio	Ref	Ref
	Have TV or Radio	1.06 (1.04, 1.09)	1.01 (0.98, 1.05)
Livelihood	Pastoralist	Ref	Ref
	Agrarian	1.31 (1.19, 1.42)	1.17 (1.06, 1.29)
Human resource at the HP	Have level 3 and below HEWs	Ref	Ref
	Have at least one level 4 HEW	0.95 (0.93, 0.97)	0.93 (0.90, 0.95)
Accessed health facility	Accessed other HF	Ref	Ref
	Accessed HP/HEW	1.13 (1.07, 1.18)	0.94 (0.87, 1.01)
Inflate variables*			
Age			0.90 ( 0.87, 0.94)
Women education	No formal education		Ref
	Have formal education		1.89 (2.12, 4.73)
Head of HH	Husband headed HH		Ref
	Wife headed HH		0.84 (0.19, 3.67)

*CI* confidence interval, *HH* Household, *HEP* health extension program, *HP* health post, *MHS* maternal health services; *HF* health facility, *HEW* health extension worker, *TV* television, *HEWs* health extension worker

*Inflate variables are variables that increases the probability of taking no services. They are selected based on literatures.

There is overlap of exposure variable (Adherence to recommendation of MOH to ANC) and intensity of CoC in terms of frequency of ANC which is included in both. We have re-analyzed the analysis by removing the overlap from the exposure variable. The result showed that there is no difference in the effect of the exposure on the outcome (Table 5).

**Table 5:** Sensitivity analysis of factors associated with intensity of continuum of care among fixed cohort of women in Ethiopia

Background characteristics		Crude IRR (95% CI)	Adjusted IRR (95% CI)
Adherence to recommendation of MOH to ANC	No	Ref	Ref
	Yes	1.29 (1.26, 1.33)	1.25 (1.22, 1.29)
Exposure to HEP	No	Ref	Ref
	Yes	1.11 (1.07, 1.15)	1.22 (1.15, 1.28)
Age		1.00 (0.99, 1.00)	1.04 (1.01, 1.08)
Women education	No formal education	Ref	Ref
	Have formal education	1.09 (1.07, 1.12)	1.05 (1.02, 1.08)
MHS awareness by husband and wife at HP	Both are not aware for all MHS	Ref	Ref
	At least one of them aware	1.01 (0.96, 1.05)	1.07 (1.01, 1.13)
	Both are aware for all MHS	1.02 (0.89, 0.98)	0.96 (0.96, 1.08)
Told at least one danger sign	No	Ref	Ref
	Yes	1.17 (1.14, 1.19)	1.14 (1.11, 1.18)
The second youngest child place of delivery	Home or other place	Ref	Ref
	Health institution	1.18 (1.15, 1.22)	1.18 (1.15, 1.21)
Wealth index	Lower quintile	Ref	Ref
	Middle quintile	0.94 (0.91, 0.97)	0.91 (0.88, 0.95)
	Higher quintile	1.03 (1.00, 1.06)	0.97 (0.93, 1.01)
Family size	≤ 4 peoples	Ref	Ref
	5–8 peoples	0.97 (0.95, 0.99)	1.01 (0.97, 1.05)
	≥ 9 peoples	0.88 (0.84, 0.92)	1.01 (0.95, 1.08)
Head of HH	Husband headed HH	Ref	Ref
	Wife headed HH	1.04 (0.98, 1.09)	0.96 (0.90, 1.03)
Number of medical equipment		1.01 (1.00, 1.01)	1.01 (1.01, 1.02)
Media	Have no TV or Radio	Ref	Ref
	Have TV or Radio	1.06 (1.04, 1.09)	1.01 (0.98, 1.05)
Livelihood	Pastoralist	Ref	Ref
	Agrarian	1.31 (1.19, 1.42)	1.19 (1.08, 1.31)
Human resource at the HP	Have level 3 and below HEWs	Ref	Ref
	Have at least one level 4 HEW	0.95 (0.93, 0.97)	0.94 (0.91, 0.97)
Accessed health facility	Accessed other HF	Ref	Ref
	Accessed HP/HEW	1.13 (1.07, 1.18)	0.95 (0.88, 1.02)
Inflate variables*			
Age			0.90 ( 0.87, 0.94)
Women education	No formal education		Ref
	Have formal education		1.89 (2.12, 4.73)
Head of HH	Husband headed HH		Ref
	Wife headed HH		0.84 (0.19, 3.67)

*CI* confidence interval, *HH* Household, *HEP* health extension program, *HP* health post, *MHS* maternal health services, *HF* health facility, *HEW* health extension worker, *TV* television, *HEWs* health extension worker

*Inflate variables are variables that increases the probability of taking no services. They are selected based on literatures

## Discussion

Our finding show that there is very high drop out of maternal health service uptake along the continuum. Even though very large proportion of women took at least one of the services, only one in seven women completed the continuum of care. Place of first ANC visit didn’t have a significant effect on the completion of CoC. Adhering to MOH recommendation of ANC visit increases the uptake of CoC. Intensity of CoC increases among women who are residing in agrarian areas, who have HEP exposure, who hear about danger sign, and who delivered their second youngest child at HF.

In spite of very large percent of women taking at least one components of COC, small percent of them completed CoC in agreement with pocket studies in Ethiopia in which only 9.7–12.1% of women completed continuum of care findings [21, 28]. Furthermore, results from 2016 DHS have indicated similar findings [34]. The findings indicate that there is much to be done to improve the uptake. Several factors related to socioeconomic, cultural and health facilities are likely to contribute more to the low completion of continuum of care.

Our result demonstrates that nearly equal proportion of women took their ANC-1 at HP and HC or higher level of facility but, it didn’t show difference in completion of CoC which is in conformity with a study conducted elsewhere in Ethiopia [35]. Those findings are in the contrary to the recommendation of the MOH in two ways: (1) HPs aren’t allowed to provide focused ANC due to lack of infrastructure and equipment; (2) HCs or other higher level HFs are expected to have better completion of CoC due to their better standards [26, 36]. The lack of difference in completion of CoC implies that there is compromised quality of care across the health facilities (HFs) because retention in care needs better quality of services. Previous studies affirmed that communities fail to complete the CoC due to unmet need of services and lack of equipment across HFs [24, 37]. Thus, compromised quality of care deter the completion of CoC. These findings imply the quality of ANC-1 service, which is the critical entry point of the women, at HC should be in question.

One in seven women completed maternal health CoC, and only a quarter of women took ANC visits as per the recommendation of MOH which is very low in contrast to the recommendations [9, 13]. ANC visits as per the MOH recommendation consistently increases the likelihood of uptake of CoC across all hypothesis testing models. Previous studies showed that higher number of ANC visits are associated with increased uptake of MHS [38, 39]. When those contacts are at the recommended type of HFs, it improves the continuity of maternity care [40]. The Ethiopian MOH recommends to provide ANC-4 at HFs other than HP due to incapability of HPs by the standard to examine pregnancy, treat complications, and attend delivery [26, 41]. These findings tells us that most women are not vising the minimum required number of ANC visits, and they are following at the health posts. This may imply that those women may not have access to higher HFs. Even though increasing number of ANC contacts is important to improve CoC, it is equally important to promote visit at higher health facilities.

This study demonstrated that exposure to HEP increases intensity of continuum of care, which is in conformity with a study conducted in Ethiopia [42]. This shows that HEP is meeting one of its targets that is improving maternal and child health [43]. This implies that a good implementation of HEP through home visit, outreach visit and enhanced quality of HP services improves the CoC. Moreover, women who heard about danger sign and women who delivered their second youngest child at health facility were more likely to have higher intensity of continuum of care. This finding is in line with the findings of previous studies in which women who received health education on maternal healthcare services were more likely to complete the continuum of care [34, 44]. It is because of a good consultation including reminding on danger signs, and persuade the users to understand their health status increases the attachment of the women with the facility. Thus, providing appropriate care at time of their contact with health facility or health professional has a potential to improve the continuum of care.

Women from agrarian areas receive a higher intensity of CoC as compared to pastoralist women. As different research shows pastoralism is a conundrum to provide health services, particularly socio-cultural factors, geographic access and service quality of health services are the major bottle necks [45, 46]. Even though those problems are also found among agrarian areas it is not arduous like in pastoralist areas. This implies that there is inequity in service availability and accessibility among agrarian and pastoralist areas in Ethiopia.

The study has two major strengths. The study conducted from nationally representative sample of HHs and it includes women from both agrarian and pastoralist regions. Moreover it sees the contribution of HEP. As limitation recall bias might be there in measuring ANC and delivery. Other limitations of the study are other variables such as distance to health facility and quality of care at different health facility which determine the completion of the CoC were not measured.

## Conclusion

Completion of maternal health continuum of care is very low in Ethiopia, however most of the women use at least one of the services. Place of first ANC visit didn’t have significant effect on the completion of continuum of care. Adherence to the MOH recommendation of ANC visit, which is at least 4 ANC visits at higher HFs, increased the likelihood of intensity of continuum of care. Despite the MOH recommendation, most women are following focused ANC visits in substandard health facilities. Moreover focused ANC visits at higher HFs didn’t have better completion of CoC as compared to focused ANC visits at HPs. Intensity of CoC is also positively associated with exposure to HEP, knowledge of danger signs, and delivery of second youngest child at health facility. Women in the pastoralist areas were at a disadvantage compared to women in the agrarian in terms of the intensity of CoC which needs further consideration. To boost the uptake of all maternal health services, it is crucial to work on quality of health facilities to retain mothers in continuum of care, and promoting adherence to MOH recommendations of ANC visit. Promoting adherence of ANC visit may need upgrading the infrastructures of HPs and increasing access of health centers to the community. For the scientific community, it is good to investigate effect of quality of ANC at different level of HFs on completion of maternal health continuum of care.

## Data Availability

The datasets used during the current study is available from the corresponding author on reasonable request.

## Abbreviations

ANC: Antenatal care
BIC: Bayesian information criterion
CI: Confidence interval
HCs: Health centers
HEP: Health extension program
HEW: Health extension worker
HF: Health facility
HHs: Households
HP: Health post
IRR: Incidence rate ratio
MHS: Maternal health services
PNC: Postnatal care
PSM: Propensity Score Matching
RR: Rate ratio
SD: Standard deviation
TV: Television
WHO: World Health Organization
ZIP: Zero inflated Poisson
